# Combining Observation and Physical Practice: Benefits of an Interleaved Schedule for Visuomotor Adaptation and Motor Memory Consolidation

**DOI:** 10.3389/fnhum.2021.614452

**Published:** 2021-02-04

**Authors:** Beverley C. Larssen, Daniel K. Ho, Sarah N. Kraeutner, Nicola J. Hodges

**Affiliations:** ^1^Motor Skills Lab, School of Kinesiology, University of British Columbia, Vancouver, BC, Canada; ^2^Brain Behaviour Laboratory, Department of Physical Therapy, University of British Columbia, Vancouver, BC, Canada; ^3^Faculty of Medicine, University of British Columbia, Vancouver, BC, Canada

**Keywords:** consolidation, action observation, implicit processes, distributed practice, spaced practice, motor learning

## Abstract

Visuomotor adaptation to novel environments can occur via non-physical means, such as observation. Observation does not appear to activate the same implicit learning processes as physical practice, rather it appears to be more strategic in nature. However, there is evidence that interspersing observational practice with physical practice can benefit performance and memory consolidation either through the combined benefits of separate processes or through a change in processes activated during observation trials. To test these ideas, we asked people to practice aiming to targets with visually rotated cursor feedback or engage in a combined practice schedule comprising physical practice and observation of projected videos showing successful aiming. Ninety-three participants were randomly assigned to one of five groups: massed physical practice (Act), distributed physical practice (Act+Rest), or one of 3 types of combined practice: alternating blocks (Obs_During), or all observation before (Obs_Pre) or after (Obs_Post) blocked physical practice. Participants received 100 practice trials (all or half were physical practice). All groups improved in adaptation trials and showed savings across the 24-h retention interval relative to initial practice. There was some forgetting for all groups, but the magnitudes were larger for physical practice groups. The Act and Obs_During groups were most accurate in retention and did not differ, suggesting that observation can serve as a replacement for physical practice if supplied intermittently and offers advantages above just resting. However, after-effects associated with combined practice were smaller than those for physical practice control groups, suggesting that beneficial learning effects as a result of observation were not due to activation of implicit learning processes. Reaction time, variable error, and post-test rotation drawings supported this conclusion that adaptation for observation groups was promoted by explicit/strategic processes.

## Introduction

There is considerable evidence that people can learn motor skills from watching others, and that it can augment physical practice [for reviews see Ashford et al. (2006), Hodges (2017), Hodges and Ste-Marie (2013), Maslovat et al. (2010a)]. However, when it comes to prescribing when to provide observational practice to optimize performance and learning, there are limited guidelines as to how observational and physical practice should be best integrated (c.f., Shea et al., 2000; Weeks and Anderson, 2000; Ong and Hodges, 2012). In addition to questions about *when* to schedule observational practice, there is debate about the mechanisms supporting *how* observational learning works and the processes which are shared or different from physical practice. In the current experiment, we investigate implicit and explicit contributions underlying observational practice effects in a visuomotor adaptation paradigm under various conditions where observational and physical practice are combined. We study both the immediate and longer-term (after a 24 h rest) consequences of combining observational and physical practice in comparison to physical practice alone, for effectively adapting to novel visuomotor conditions.

Researchers have explored methods of practice that may augment or even substitute for physical trials. An overabundance of physical exposure to a repetitive task may be impractical as there is an increased potential for injury or fatigue (Fry et al., 1991). Physical practice might also be limited before practice begins (e.g., in clinical populations) and it is more costly than methods such as watching demonstrations or rehearsing mentally, which do not require direct exposure to equipment. One popular applied practice method involves the inclusion of demonstrations as part of a training block to serve as an adjunct or replacement for physical practice. Although learning through observation is effective, it is rarely as effective or more effective than physical practice, failing to engage the same processes which would be needed to change behavior in the short and long-term (Hodges et al., 2007; Maslovat et al., 2010b; Ong and Hodges, 2010; Trempe et al., 2011). It is therefore important to consider what processes are shared or different between observational and physical practice and then to determine how these might be optimized through practice methods where demonstrations and physical practice are combined (e.g., Deakin and Proteau, 2000; Shea et al., 2000; Ong et al., 2012).

When learning to adapt movements in novel environments, participants improve after both physical practice and observational practice (e.g., Mattar and Gribble, 2005; Ong and Hodges, 2010; Larssen et al., 2012; Ong et al., 2012). Adaptation as a result of physical practice is thought to involve both implicit and explicit learning processes. Implicit processes are proposed to operate largely outside of conscious awareness and are impervious to instructions (e.g., Mazzoni and Krakauer, 2006; McDougle et al., 2015). The predominant hypothesis is that implicit adaptation processes are modulated by the detection of errors between actual visual feedback and predicted feedback associated with congruence between actions and their anticipated effects [yet see Hadjiosif et al. (2020)]. When there is a conflict between movement outcome and efference-based predictions about this feedback, this conflict causes the motor system to adapt an implicit, internal map of relative space (Cunningham, 1989; Tseng et al., 2007; Haith et al., 2016). Behavioral evidence in support of this implicit process is witnessed immediately following physical practice when the novel environment is returned to normal. Even though people are aware that conditions have changed, unintentional errors, or “after-effects” are seen in the opposite direction of the imposed rotation or force (e.g., Redding and Wallace, 1996; Redding et al., 2005; Lei et al., 2019). These after-effects are thought to alert to an implicit recalibration of the sensorimotor system (e.g., Ruttle et al., 2016; Modchalingam et al., 2019), providing a true indication of “motor” learning (Redding and Wallace, 1993; Frensch, 1998; Krakauer et al., 2000; Taylor and Ivry, 2012). Explicit processes are described as being available to consciousness and drive change through implementation of deliberate aiming strategies (e.g., McDougle et al., 2016). Alerting participants to the nature and direction of a perturbation or providing an aiming strategy are methods often used to encourage adaptation by explicit means, often characterized by longer planning time and increased variability in aiming errors early in practice (e.g., Mazzoni and Krakauer, 2006; Benson et al., 2011; McDougle et al., 2016).

The contributions of implicit and explicit processes supporting adaptation learning through observation are debated. Some of the debate appears to be dependent on the type of adaptation task as well as how implicit/explicit processes are assessed. For example, a secondary motor task performed simultaneously with observation of an actor adapting to a force perturbation impaired adaptation for observers (Mattar and Gribble, 2005). Impairments were not seen when the secondary task was purely cognitive. This led to the conclusion that adaptation via observation was in part driven by implicit activation of the observer's motor system, potentially engaging motor simulation processes (Gallese, 2009). However, motor secondary tasks can also interfere with other processes that help later motor memory recall, such as imagery, questioning the supposed involvement of the motor system for observational practice [see Di Rienzo et al. (2015), Di Rienzo et al. (2016), Eaves et al. (2016)]. The motor simulation hypothesis of action observation is based on neurophysiological evidence of an observation network (or “mirror neuron system”) in the human brain, which activates during both movement execution and observation (e.g., Rizzolatti et al., 1996; Rizzolatti and Craighero, 2004; Fogassi et al., 2005; Buccino et al., 2006). Activation of this network is dependent on the motor experiences of the observer (Calvo-Merino et al., 2005, 2006, 2010). Therefore, it is surprising that when watching a novel action, without prior physical practice, that observational practice would activate this motor network, rather than encouraging the formation of visual representations (Adams, 1987; Carroll and Bandura, 1990) associated with a more explicit/strategic process of adaptation (Maslovat et al., 2010b).

In studies of visuomotor adaptation, where the learner is required to learn a novel relationship between their actual hand movements and the adapted (rotated) movements of a cursor, evidence against the idea that observational learning is an implicit, motor driven process has been presented (e.g., Ong and Hodges, 2010; Ong et al., 2012). Here observers show direct performance benefits associated with watching a partner move in an altered environment, but unlike physical practice participants, do not show sensorimotor after-effects. This absence of after-effects has been attributed to the absence of an implicit, movement-based error signal (i.e., discrepancy between the actual visual feedback and predicted sensory consequences associated with moving or simulating another's movement). Rather, performance gains for observers have been linked to explicit, strategic processes, associated with improved awareness about the imposed rotation, in comparison to physical practice participants, as well as other measures suggestive of strategic adjustments (such as longer reaction times and increased trial-to-trial variability; Hinder et al., 2010; Benson et al., 2011; Ong et al., 2012).

Combining physical practice with observation may be one method that could bring about motor simulation during action observation because individuals have experiences which are expected to activate motor areas during observation. In one study, learners who engaged in observational practice augmented with some intermittent physical practice, were more accurate than a 100% physical practice group during acquisition and also showed larger after-effects (Ong et al., 2012). However, observers in this study were also encouraged to engage in imagery and to predict the hand trajectory of the model on cursor-only trials, as well as estimate their own hand trajectories on physical practice trials. It is unclear which variable or combination of variables was responsible for the subsequent adaptation effects. In a second study, where physical practice was only provided before observational practice, not interspersed (there were no imagery and trajectory estimation trials either), after-effects did not increase after observing (Lim et al., 2014). Therefore, it might be the case that interspersing observation with physical trials reinforced the specific learning processes associated with each type of practice and neutralized the shortcomings of either method on its own. These various methods of combining observational and physical practice (i.e., blocked or interspersed) have not been compared in a single study where other difference variables are controlled. Moreover, only short-term adaptation processes have been studied and not retention effects, which would indicate any memory consolidation benefits associated with these combined methods of practice. For visuomotor rotation tasks, there is evidence that consolidation may take up to 24 h (Caithness et al., 2004; Trempe and Proteau, 2010).

In non-adaptation tasks, the amount of time that elapses between physical practice trials and observational practice trials appears to play a role in enhanced consolidation. For example, in finger tapping tasks, a period of observation immediately after physical practice benefitted later retention (Zhang et al., 2011) and providing observation concurrent with physical practice, or at least in immediate succession, was shown to be beneficial for longer-term retention (Bove et al., 2009). Recently, Moore et al. (2019) compared individuals learning a tracking task by either physical practice alone or interleaving observation trials with 60% physical practice. Despite less physical practice, this latter group did not differ from the physical practice group in a 24 h and 1-week retention test, but neither were there retention benefits.

In the following experiment, we tested various methods of scheduling observation and physical practice to determine what type of schedule (i.e., timing of observational practice) is best for immediate and longer-term retention in a novel visuomotor adaptation task. Our primary interest was to determine if and how combined schedules of observation and physical practice impacts the presence and magnitude of unintentional after-effects (used to infer the extent to which implicit recalibration of the sensorimotor system has occurred). We compared groups that received combined practice; including bouts of observational practice before, after, or interspersed with physical practice, to two physical practice only groups. If observation is a key component to maximizing what is learned during physical practice, interspersed demonstrations throughout practice would be most beneficial to measures of long-term learning (i.e., retention) in comparison to blocked schedules of physical practice and demonstrations. If interspersing demonstrations with physical practice is able to activate simulation-type processes associated with learning by doing, we expected that physical practice intermixed with observational practice would generate a stronger implicit learning response (i.e., greater after-effects) than that brought about by only physical practice (matched to the number of physical practice trials for the combined groups) or observation given only after or before physical practice. The two physical practice groups were matched to the combined groups for either the amount of physical practice or the amount of total practice (physical and observation combined). Importantly, the group matched for physical practice only, underwent a spaced practice protocol, to control for distributed practice benefits which might accrue from small periods of rest between physical practice trials (Bönstrup et al., 2020).

## Methods

### Participants

Ninety-three (*n* = 18–19/group), right-handed volunteers (self-reported and confirmed through the Edinburgh Handedness inventory, Oldfield, 1971) from the University community (*M* age = 23 yr, *SD* = 5.6; *F* = 68) participated^1^. They were pseudo-randomly assigned to one of five groups. There were three combined practice groups: a pre-practice group (Obs_Pre, *n* = 19) that engaged in action observation practice before physical practice; a post-practice group (Obs_Post, *n* = 18) that completed physical practice before observation; and an interspersed group (Obs_During, *n* = 18) that alternated between observational and physical practice. Massed (Act, *n* = 19) and distributed (Act+Rest, *n* = 18) physical practice only groups were also included for comparison. Issues with data processing, failure to complete all testing or adhere to instructions resulted in slightly fewer participants than the desired *n* = 20/group. Our inclusion criteria required participants to report normal or corrected-to-normal vision with no known neurological deficits. All participants were naïve to the task and purpose of the study and provided written informed consent. All procedures were approved by the research ethics' board of the University.

### Task and Apparatus

A PC (Dell Inspiron 531, AMD Athlon^TM^ 64 × 2, 5,600+, 2.9 GHz dual core processor) was used to run a custom aiming task that was programmed using LabVIEW^TM^ software (version 9.0, 2009). Participants executed reaching movements using a computer mouse to maneuver a cursor, within a digitized display, from a stationary starting position toward one of five possible targets. Participants sat in a chair facing a horizontal, semi-silvered mirror, fixed 30 cm above a graphics tablet (Calcomp Drawing Board VI, 200 Hz) which measured 2D displacement. An inverted computer monitor (ViewSonic E70f–CRT 17 inch monitor, 1,280 × 1,024 resolution, refresh rate: 66 Hz), projected an image of the visual stimuli (start position and aiming targets) and cursor position onto the mirror, situated 30 cm above the mirror. The cursor was represented by a circular marker of 0.4 cm diameter and controlled by a mouse attached to a custom-made plastic extension with cross-hairs for placement of the right index finger. The room was darkened and a chin rest was positioned in front of the equipment to ensure full vision of the projected image only. The visual stimuli included the central white start square and five radially arranged targets that were presented 9.5 cm from the start square. Targets were located at 0, 72, 144, 216, and 288° along the clockwise direction.

Within a block of 5 trials, each of the five targets was presented in a pseudo-random order and when physically performing, participants were required to aim fast and accurately to make shooting movements through the target (e.g., Tseng et al., 2007; Huang and Shadmehr, 2009). Participants were also instructed to generate straight, uncorrected trajectories while aiming past each target. On trials where movement times surpassed 350 ms, the experimenter verbally prompted the participant to move faster on the next trial (these trials were not excluded from analysis). The movement time (MT) constraint was to ensure that participants were not making online movement corrections. No restrictions on reaction time (RT) were imposed (i.e., the interval between target onset and movement initiation), but RTs were measured to give an indication of movement preparation associated with more strategically planned, between-trial adjustments (Hinder et al., 2010; Benson et al., 2011; Ong et al., 2012). After reaching past the target, the trace of the participant's cursor trajectory remained on the screen for 1 s. Participants were instructed to return to the start square once their feedback disappeared to initiate the next trial. Upon returning to the start position, cursor vision was prevented until the cursor entered within a 4.75 cm radius from the origin. The next trial began (as indicated by a new target appearing), 2 s after return to the start.

For the observational practice trials, participants viewed a video of an experienced (accurate) actor performing the adaptation reaching task (MT, *M* = 238 ms, *SD* = 19 ms; CE at peak velocity, *M* = 0.19 deg, *SD* = 3.3 deg). Performance of 50 trials was recorded with a web camera (Logitech Quickcam Pro 9000) that was mounted above the actor's head just underneath the projection monitor, such that the video was able to detect the actor's lower arm and hand movements and the resultant cursor path feedback while aiming in a 30° clockwise (CW) rotated environment. A panel of white light-emitting diodes (LEDs), fixed to the underside of the semi-silvered mirror permitted vision of the actor's hand through the mirror during filming. During observation trials, participants were still seated in front of the mirror-box apparatus and watched a mirror-reflected image of the video in the same plane of action as required during physical practice.

### Procedure

**Figure 1:**
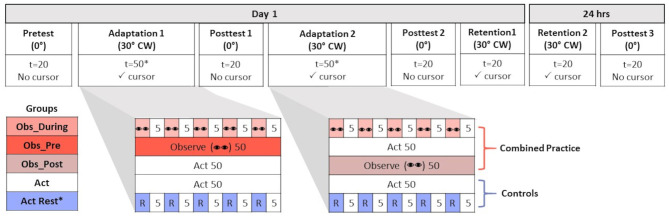
Table of progression of experimental procedures across all experimental conditions. Participants either performed in a normal (no rotation, 0°) or new environment [30° clockwise (CW) cursor feedback rotation]. The number of trials (t) is reported for all conditions. The number of trials (t) is reported for all conditions. Conditions where visual cursor feedback was (✓ cursor) and was not present (No cursor) is reported. Groups differed in terms of the practice schedule they received during Adaptation 1 and Adaptation 2. Combined practice groups received a combination of observation (represented by “eyes”) and physical practice (Act) that was either interleaved in an alternating schedule (Obs_During = 5 trials of observation + 5 trials of Act) or blocked (Obs_Pre = 50 trials Observe + 50 trials Act; Obs_Post = 50 trials Act + 50 trials Observe). Two control groups received only physical practice in either a massed (Act) or spaced schedule interleaved with rest (*R* = 1 min). All groups experienced 50 trials of either observation, physical, or combined practice during each Adaptation time point (100 trials total), with the exception of Act+Rest (*25 trials at each time point).

The experiment was divided into 8 phases over 2 days of testing: Pre-test, Adaptation 1, Post-test 1, Adaptation 2, Post-test 2, immediate retention (Retention 1), 24 h delayed retention (Retention 2), and Post-test 3 (see Figure 1). Pre-tests and Post-tests were performed in known, normal, non-rotated environments, whereas adaptation and retention tests were performed in known novel (rotated) environments. Moreover, participants underwent different conditions of practice during the Adaptation phases, depending on group, whereas retention tests were always the same for all groups involving physical practice only after a short (immediate retention) or long (delayed retention) rest. On day 1, participants were first allowed to familiarize themselves with the overall task parameters by aiming in a normal (veridical) environment in which the cursor path corresponded directly with hand movements. Vision of cursor position and target location were both provided during 20 familiarization trials. Following familiarization, participants engaged in a pre-test (*t* = 20) whereby aiming continued to occur in a veridical manner; however, no feedback was provided in this phase (of either their hand or the cursor trajectory relative to the target). This proprioceptive reaching pre-test provided a reference for determining after-effects in subsequent post-tests performed under the same conditions.

Before commencing each phase, participants were made aware of the visuomotor conditions that they would experience. For the normal environment (no rotation), the participants' goal was to direct the cursor toward the target using their index finger. While in the normal environment, the perimeter of the workspace was highlighted with a blue border to serve as an additional visual contextual cue. During the adaptation phase, in which the cursor trajectory was rotated 30° CW relative to hand movements, participants were told that the environment had been changed, compared to the normal condition, and the response of the cursor was altered. There was now no colored border around the workspace. Despite the novel aiming environment, participants' goals remained the same as in the pre-test. In order to successfully acquire the target, participants needed to aim their index finger 30° counterclockwise (CCW) relative to the actual target position (though this strategy was not conveyed).

Practice in the rotated environment was divided into 2 adaptation phases (Adaptation 1 and Adaptation 2). With the exception of the Act+Rest group that received a distributed schedule of rest and physical practice (*t* = 50), all other groups received the same number of total practice trials (*t* = 100) in the 30° CW-rotated environment (presented as either only physical practice, or combined observational, *t* = 50 and physical practice, *t* = 50). After completing either 25 (Act+Rest) or 50 trials of their respective practice conditions, all participants completed an initial test of after-effects in a normal environment (i.e., no feedback; Post-test 1, *t* = 20). Participants then resumed their respective practice schedules depending on their group assignment. The Obs_Pre group first received 50 trials of observational practice during Adaptation 1 before physically practicing for 50 trials in the rotated environment during Adaptation 2. The Obs_Post group completed 50 physical practice trials with the 30° CW rotation in Adaptation 1 before watching the 50 observation trials (Adaptation 2). The Obs_During group alternated between five observational practice trials and five physical practice trials until two adaptation phases of 100 total trials were concluded. The Act group completed two phases of 50 physical practice trials each, and the Act+ Rest group completed two phases of 25 physical practice trials each, with 1 min rest after each 5 trial block of physical practice.

Immediately at the end of adaptation, a second test of after-effects was conducted (Post-test 2, *t* = 20). This was followed by a short 1 min rest after which participants were returned to the rotated environment for an immediate retention test (Retention 1, *t* = 20) in the CW rotated environment with visual feedback. After ~24 h interval, participants returned to complete a second retention test (Retention 2; *t* = 20) and a final test of after-effects (Post-test 3, *t*=20). At the end of testing on day 2 and before debriefing, participants completed a drawing test probing their explicit awareness of the rotation, including its size and direction. Each participant was presented with a paper diagram displaying the 5 targets relative to the central start position. They were asked to draw where their hand would have moved (i.e., planned aiming trajectory) in order to successfully aim the computer cursor along the desired trajectory to hit each of the 5 targets under the novel environment aiming conditions (i.e., Adaptation and Retention). The angle between the planed aiming trajectory of the hand relative to desired cursor trajectory for accurate performance was used to calculate perceived aiming angle.

### Performance Analysis

Calculation of participant movement kinematics (used to determine spatial errors) was performed using a custom LabVIEW^TM^ program (version 9.0). Movement onset was defined as the time when the cursor left the home square and movement end was the time when the cursor exceeded the 9.5 cm radius of the target array (allowing calculation of RT and MT). Aiming trials where movement times (MT) exceeded 1,000 ms were excluded from analyses. This resulted in a mean exclusion of <0.8% of the total trials (Obs_Pre = 0.7%, Obs_Post = 0.8%, Obs_During = 0.6 %, Act = 0.5%, Act+Rest = 1.7%). Mean directional constant radial error (CE; in degrees) was our primary measure and this was calculated for each trial and reported as a mean for each 5-trial block (based on all 5 targets). Mean CE is the angle between the reference trajectory joining the center (i.e., home position) and the intended target and the trajectory joining the center and the actual cursor position. This was measured at peak tangential velocity to ensure that errors reflected motor planning not feedback based control (e.g., Bernier et al., 2005; Larssen et al., 2012). A positive value for error denotes a CW error whereas a negative value represents a CCW error. A positive error was the result of an under-correction to the 30° cursor rotation, whereas negative errors indicated an over-correction.

Variability in aiming errors (Variable Error, VE) was calculated during Adaptation 1 and 2 based on the standard deviation (SD) of CE for each block of 5 consecutive trials for each participant. Mean RTs were calculated in a similar manner, based on individual means for each 5-trial block. RT was characterized as the difference between target onset and movement onset. Both VE and RT data were supplemented with descriptive statistics regarding the rotation awareness test given at the end of practice, to facilitate conclusions about the type of control strategies governing performance.

### Statistical Analysis

Performance metrics related to initial adaptation (Adaptation 1 and 2), learning and savings over the 24 h consolidation interval, and after-effects, were evaluated using separate linear mixed effects (LME) models, with the lme4 package (Bates et al., 2015) in R version 3.2.4 (R Development Core Team, 2013). LME models are almost identical to more traditional fixed-effects ANOVA, except that they include all trials as separate observations for each participant and allow testing (and hence control) of both fixed and random (subject) effects, especially suited to RM designs (Galwey, 2006). All models were used to assess error as a function of group, time point, their interaction, and a random intercept for each participant and are reported in reference to the Act group (see Supplemental Materials for tables of LME outputs).

Separate LME models were conducted on the CE data to probe adaptation during Adaptation 1 and 2 based on the same 5 physical practice blocks which were common to all groups: blocks 2, 4, 6, 8, 10. Note there were four groups/time point as there were no data for the Obs_Pre and Post groups in Adaptation 1 and 2, respectively. To investigate savings (comparing early adaptation and delayed retention) and any gains or losses following the delayed retention interval (comparing late adaptation and 24 h retention test), a LME model test was conducted that included the first adaptation time point (first five trials where participants physically practiced), as well as Retention 1 (last 5 trials; Day 1) and Retention 2 (first 5 trials; Day 2) time points. To compare differences in after-effects, a LME model was run using Pre-test, Post-test 1, Post-test 2 and Post-test 3 as time points (all data were compared relative to pre-test). The same LME model design that was used for CE data during adaptation was applied to the VE and RT data during Adaptation 1 and 2.

Where relevant, between group differences across Adaptation and Retention tests were followed up with Tukey's HSD *post-hoc* tests, using the multcomp package in R (Bretz et al., 2016). Effect sizes (Cohen's *d;* Cohen, 1988) were included to characterize the magnitude of forgetting between Retention 1 and Retention 2 (as errors were shown to increase), savings from Adaptation 1 to Retention 2, and the magnitude of after-effects calculated as the difference between pre-test and each post-test (resulting in 3 separate effect sizes per group).

To establish if variability of aiming errors during Adaptation was related to subsequent after-effect magnitude, two separate omnibus *post-hoc* Pearson correlation coefficients were conducted. One on mean VE during Adaptation 1 and after-effect magnitude (absolute value of the mean difference in CE error between the last 5 trials of Pre-test and first 5 trials of Post-test 1; 4 groups), and a separate correlation for mean VE during during Adaptation 2 and after-effect magnitude (absolute value of the mean difference in CE error between the last 5 trials of Pre-test and first 5 trials of Post-test 2; 4 groups).

## Results

We first present the adaptation data, for CE, VE and RT before presenting CE data only pertaining to retention/savings and after-effects. LME outputs for all analyses are presented in (Supplementary Tables 1–5).

### Adaptation

#### Constant Error (CE)

**Figure 2:**
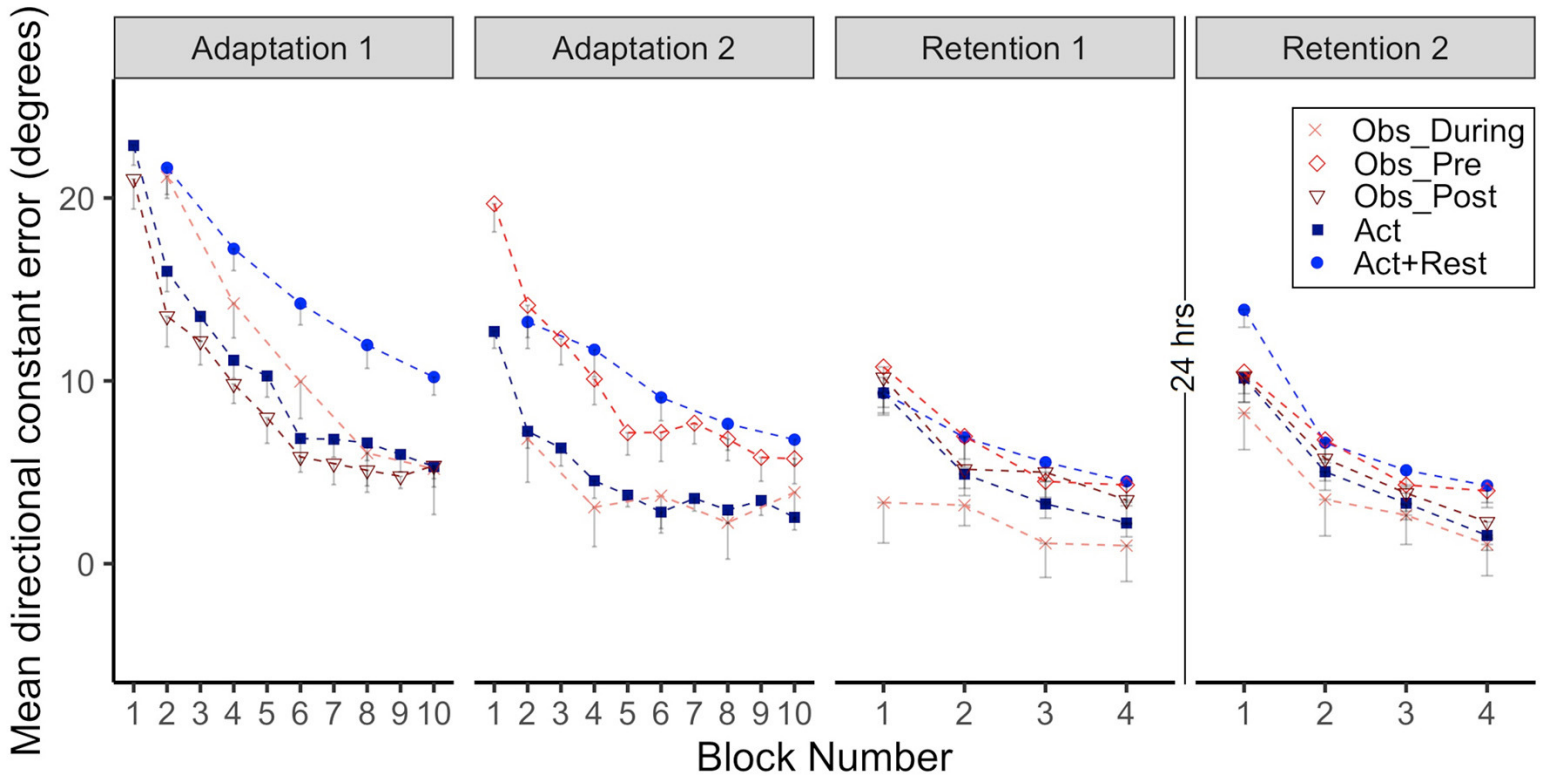
Group mean directional constant error in degrees is plotted as a function of block, where each block represents the average error of 5 consecutive movement trials. Error bars represent standard error of the mean. Data is presented for all time points where participants physically practiced aiming with rotated cursor feedback (Adaptation 1, Adaptation 2, Retention 1, and Retention 2) Positive values indicate error where the participant's cursor missed in the clockwise direction relative to the target. Due to their interleaved schedule of practice during Adaptation 1 and Adaptation 2, the Obs_During group only have means reported for block 2, 4, 6, 8, 10. We have illustrated the data for the Act+Rest group in the same way to aid visual comparison, as this group was matched to have the same practice and trial spacing as the Obs_During group. Note that statistical analyses of Adaptation 1 and 2 performance were performed on the data in blocks 2, 4, 6, 8, 10 as illustrated in the figure for all groups. Comparisons between the last block of Retention 1 and first block of Retention 2 were made to characterize offline learning/forgetting. Comparisons between the first block of adaptation practice (either Block 1, Adaptation 1, for the Act and Obs_Post groups, Block 2 for the Act+Rest, and Obs_During groups or Block 1, Adaptation 2 for the Obs_Pre group) and first block of Retention 2, were made to characterize savings.

CE data for all groups when performing in the CW rotated environment is presented in Figure 2, the first two panels show Adaptation 1 and 2 and the last two panels show immediate Retention 1 (same day) and delayed Retention 2 (after 24 h). Note how the Obs_During and Act+Rest groups only had physical practice data every other trial block during Adaptation 1 and 2. These alternate data blocks were therefore used for all statistical analyses involving adaptation.

As illustrated in Figure 2, all groups improved during Adaptation 1, this was confirmed by significant block effects, where blocks 4, 6, 8, and 10 were all different than block 2 (all *p*s < .01). There was also a significant interaction between the Obs_During group and block 8 and 10, which started out (at block2) significantly different from the Act group, but was no longer different at the end of Adaptation 1 (*ps* < 0.01). From inspection of the graphs in Adaptation 1, the groups that received massed, 100% physical practice at this stage (Act and Obs_Post) performed with less error than the groups that had spaced and less frequent physical practice (Act+Rest and Obs_During). This was confirmed by a main effect of group for the Act+Rest and Obs_During groups when compared to the Act group (*ps* < 0.01). *Post-hoc* testing showed that these two groups were not different to each other.

With regards to Adaptation 2, all blocks were again different than Block 2 (*ps* < 0.05). There were significant Group effects for Obs_Pre (*p* = 0.001) and Act+Rest (*p* = 0.004), which performed with more error than the Act group. Importantly no differences were observed between the Obs_During and Act group that had received twice as much physical practice. The interaction was only significant for Obs_Pre at Block 10 (*p* = 0.04), due to a large difference between these groups at Block 2, which was reduced by Block 10. *Post-hoc* testing showed that the Obs_During group had lower error than the Obs_Pre group (*p* = 0.004) and the Act+Rest (*p* = 0.01) group. This suggests that, at least in acquisition, observation had a benefit which was not merely a spacing effect.

#### Variable Error

**Figure 3:**
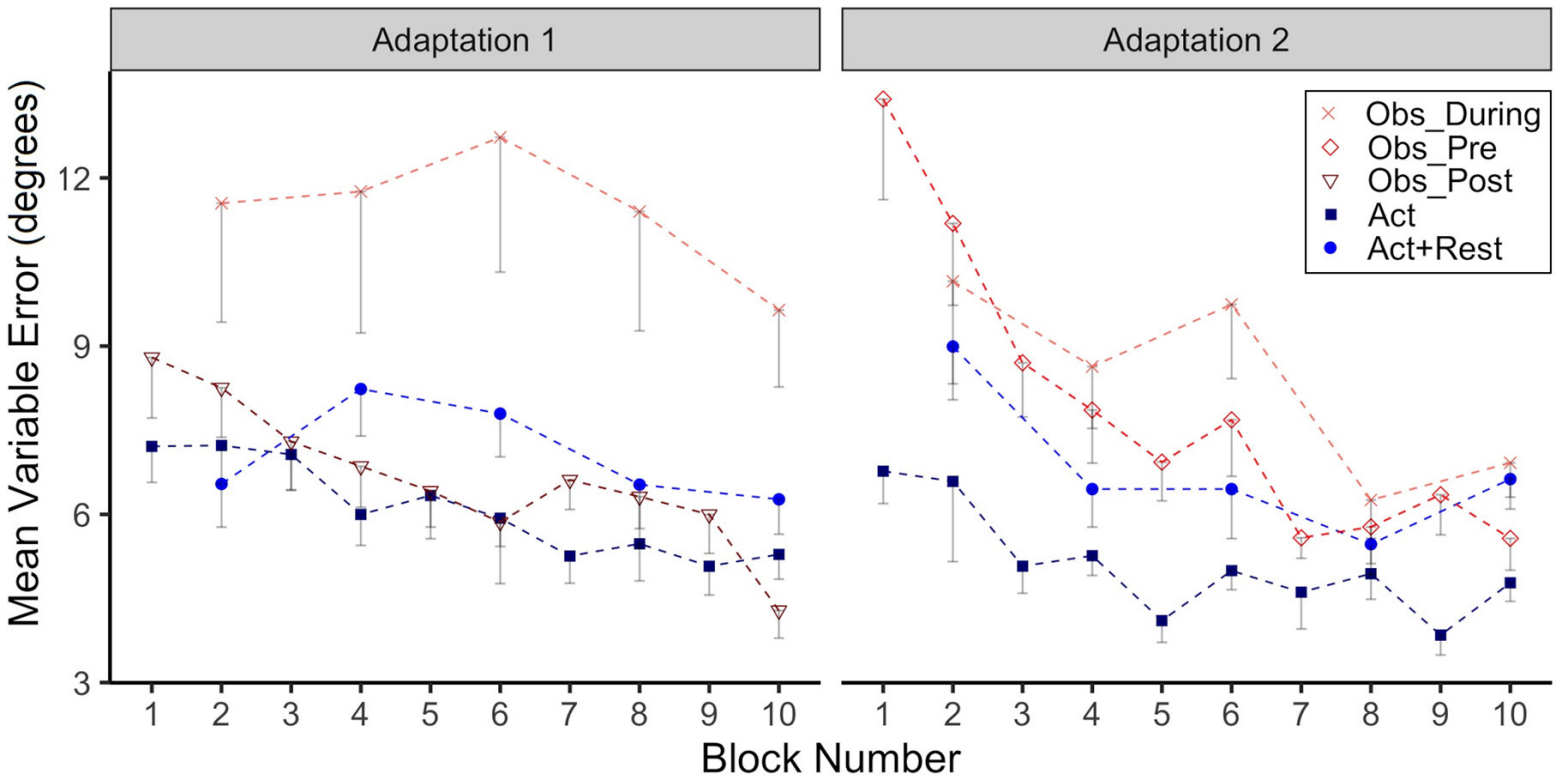
Group mean Variable Error (VE, in degrees) across Adaptation 1 and Adaptation 2 is reported as a function of block. Each block represents the average standard deviation of CE from 5 consecutive movement trials. Data is presented for all time points where participants physically practiced aiming with rotated cursor feedback. Error bars represent standard error of the mean. Due to their interleaved schedule of practice during Adaptation 1 and Adaptation 2, the Obs_During group only has means reported for block 2, 4,6, 8, 10. We have illustrated the data for the Act+Rest group in the same way to aid visual comparison, as this group was matched to have the same practice and trial spacing as the Obs_During group.

In Figure 3 we have plotted group VE as function of adaptation block for Adaptation 1 and Adaptation 2. There was high variability for the Obs_During group during Adaptation 1, confirmed by a significant main effect of group (*p* = 0.01), when comparing this group to the Act group. *Post-hoc* testing also confirmed that the Obs_During group had higher VE than the Act+Rest group (*p* = 0.03). No other group comparisons were significantly different. In Adaptation 2, both the Obs_Pre (*p* < 0.001) and Obs_During (*p* = 0.006) groups had higher VE compared to the Act group. Only the interaction was significant for the Obs_Pre group at block 8 and 10 (*p*= 0.02), reflecting the reduction in VE for this group relative to the Act group, whose variability did not change.

#### Reaction Time

**Figure 4:**
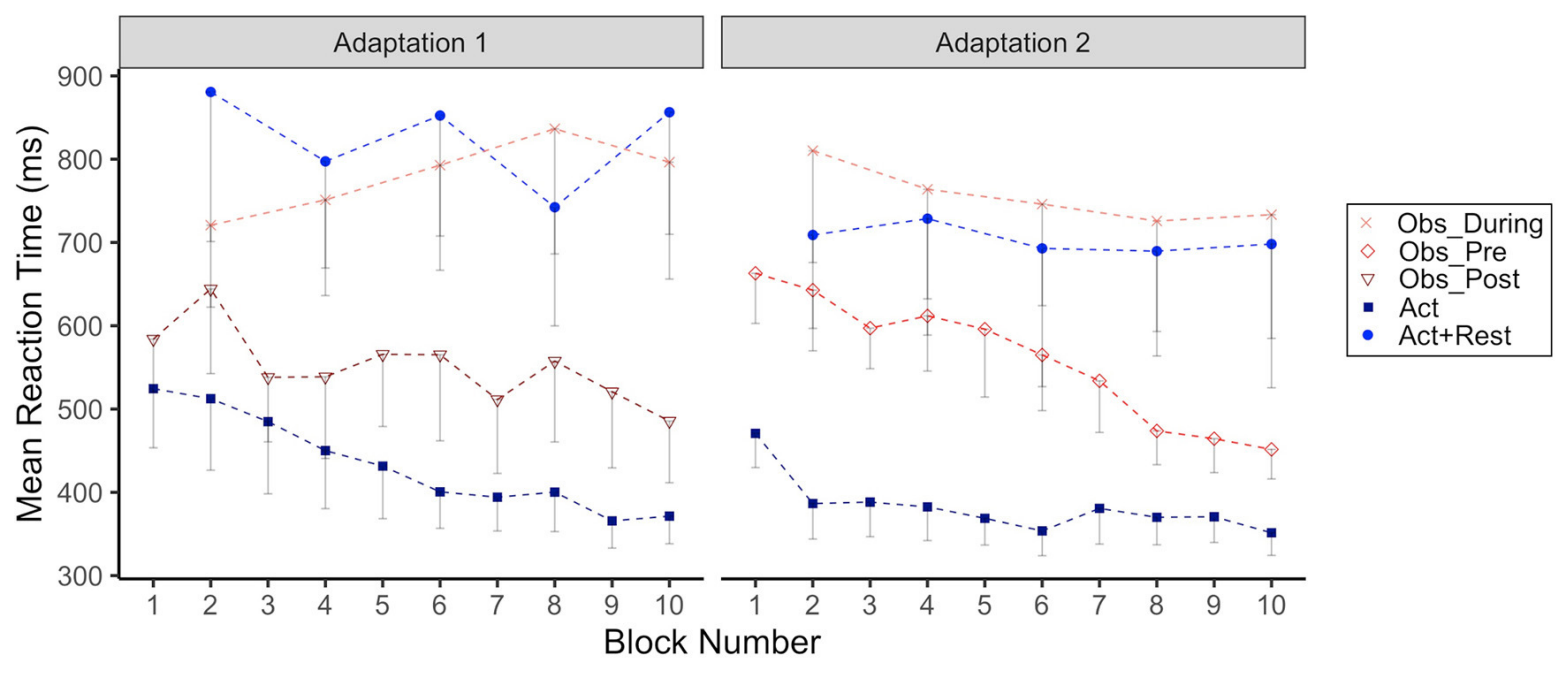
Group mean reaction time (RT, ms) across Adaptation 1 and Adaptation 2 as a function of block. Each block represents the average RT of 5 consecutive movement trials. Data is presented for all time points where participants physically practiced aiming with rotated cursor feedback. Error bars represent standard error of the mean. Due to their interleaved schedule of practice during Adaptation 1 and Adaptation 2, the Obs_During group only has means reported for block 2, 4,6, 8, 10. We have illustrated the data for the Act+Rest group in the same way to aid visual comparison, as this group was matched to have the same practice and trial spacing as the Obs_During group. Group mean RT during Adaptation 1/Adaptation 2: Obs_During (764.2/755.8 ms), Obs_Pre (not applicable/559.9 ms), Obs_Post (543.7 ms/not applicable), Act (433.6/382.3 ms), Act+Rest (766.0/681.1 ms).

In Figure 4 we have plotted group RTs as a function of adaptation block for Adaptation 1 and 2. What can be seen from this figure is that both observation and rest resulted in noticeably longer RTs compared to continuous physical practice without breaks or observation periods, across both Adaptation phases. The group effect was only statistically significant for the Act+Rest group (*p* = 0.01) compared to Act during Adaptation 1. However, both Act+Rest (*p* = 0.03) and Obs_During (*p* = 0.003) groups were different than Act during Adaptation 2. RTs showed a gradual increase for the Obs_During group during Adaptation 1, whereas a trend for decreasing RTs across blocks was noted for the groups that only had pure physical practice in this phase (Act and Obs_Post). This trend was supported by a significant interaction for only the Obs_During group at Blocks 6, 8, and 10 compared to the Act group (*p*s < 0.05*)*. In Adaptation 2, the Obs_Pre group that was now only engaging in physical practice, showed a noticeable decrease in RTs across blocks, showing RTs more in line with the Act group by the end of this practice phase. This observation was supported statistically by a significant interaction with Blocks 8 and 10 for this group only (*p*s < 0.05).

### Retention Savings and Forgetting

For CE, we compared all groups relative to the Act group across three timepoints; the first 5 trials of adaptation day 1 compared to the last 5 trials of Retention 1 and the first 5 trials of delayed Retention 2. A significant effect of timepoint for Retention 1 illustrates that all participants performed with less error at the end of day 1 compared to when they were first provided with physical practice in early adaptation (*p* < 0.01). A significant interaction was observed between the Obs_Pre group and Retention 1, due to differences between the Obs_Pre and Act groups in early Adaptation, but not in immediate retention (*p* < 0.01). The Obs_Pre group had lower initial error than the Act group on the first trials of acquisition, presumably as a result of the preceding 50 observation trials. *Post-hoc* testing confirmed that groups were not different during the first 5 trials of Adaptation on day 1 nor during the last 5 trials of Retention on Day 1 (*p*s > 0.05).

There was evidence of savings across the 24-h retention interval, supported by the significant effect of time point for Retention 2 relative to initial practice (*p* < 0.001). The only group that did not reduce errors from Adaptation 1 to Retention 2 to the same extent as the Act group was the Act+Rest group (as evidenced by an Act+Rest group X Retention 2 interaction, *p* < 0.01). *Post-hoc* testing of Retention 2 showed that only the Obs_During group was less errorful than Act+Rest group (*p* < 0.05), there were no other group differences. In Table 1 we have presented effect sizes characterizing the magnitude of savings as well as degree of forgetting across the 24-hr retention interval (there were no gains across the retention interval). With respect to savings, all groups showed large effect sizes for all comparisons (*d*s = 0.90 to 1.70; Obs_Pre, *d* = 0.70). From Retention 1 to Retention 2, moderate and large negative effects were observed, but these were smallest for the combined practice groups, representing the least amount of “forgetting” (*d* = −0.61 to −0.82), in comparison to the pure physical practice groups (*d* = −1.25 and −1.37).

**Table 1:** Effect sizes (Cohen's *d*) characterizing the magnitude of forgetting (Retention 1 minus Retention 2), savings in performance error (Adaptation 1 minus Retention 2), and after-effect magnitude (Pre-test minus Post-test 1, 2, and 3; three separate effect sizes).

	**Forgetting (Ret 1–2)**	**Savings (Adapt 1–Ret 2)**	**After-effects (Pre–Post-test 1)**	**After-effects (Pre–Post-test 2)**	**After-effects (Pre–Post-test 3)**
Obs_During	−0.61	0.97	2.06	2.36	1.71
Obs_Pre	−0.67	0.70	0.25	2.59	2.42
Obs_Post	−0.82	1.01	3.48	1.90	3.04
Act	−1.25	1.67	3.20	3.75	2.90
Act+Rest	−1.37	0.93	2.35	2.99	2.89

*All effect sizes were calculated using within-group pooled SD*.

### After-Effects

**Figure 5:**
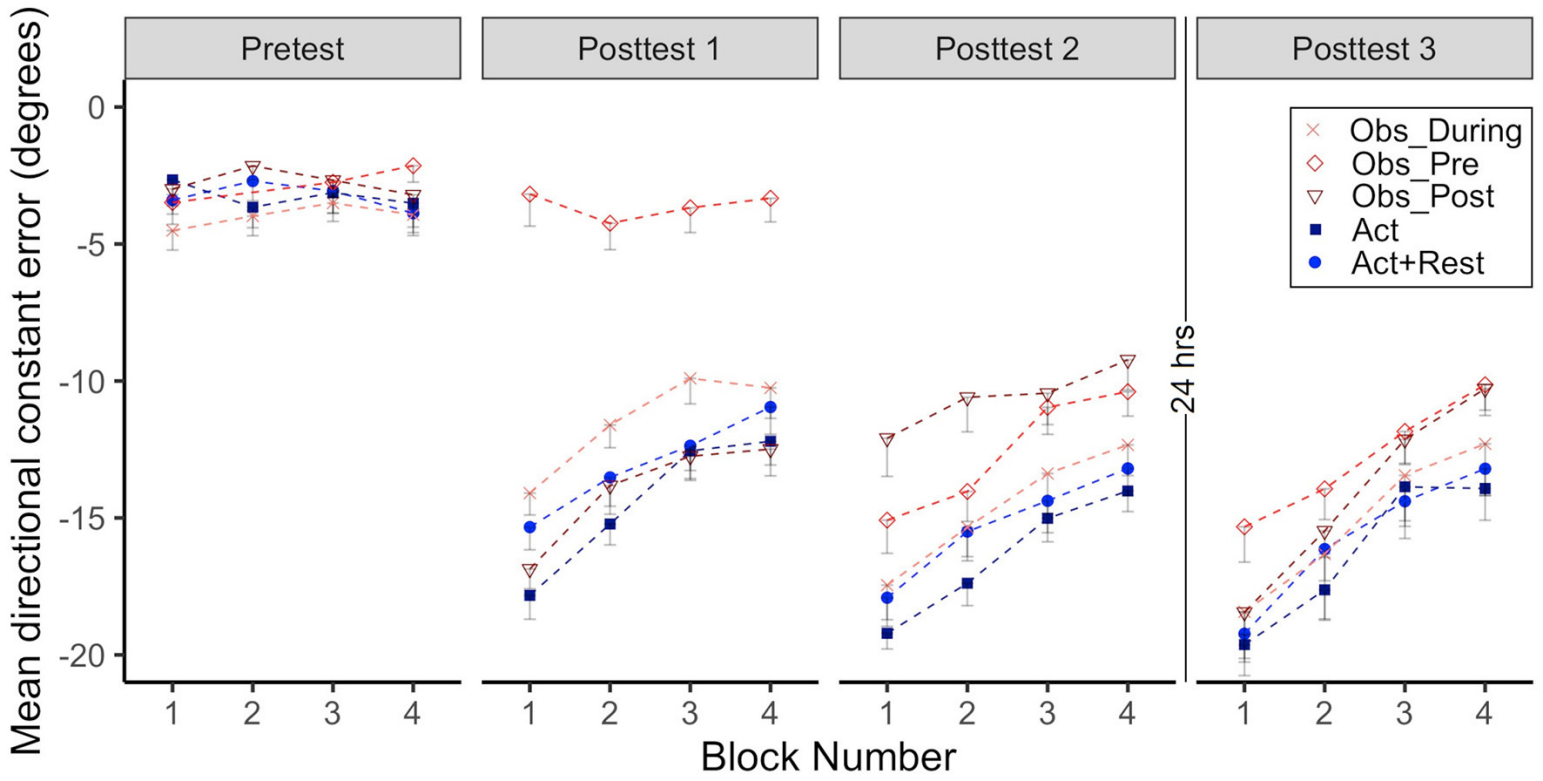
Group mean directional constant error in degrees is plotted as a function of block, where each block represents the average error of 5 consecutive movement trials. Error bars represent standard error of the mean. Data is presented for all time points where participants physically practiced aiming in a “normal” (no rotation) environment without cursor feedback (Pre-test, Post-tests 1–3). Negative values indicate error where the participant's cursor missed in the counterclockwise direction relative to the target (opposite to the direction of the rotated cursor feedback experienced in Adaptation and Retention).

To determine processes underpinning adaptation and learning effects, particularly whether a lack of difference in acquisition between the Obs_During and the Act groups could be explained by similar or different (implicit) processes, we analyzed post-test after-effects. Mean CE for all groups across the four normal environment conditions is shown in Figure 5 (Pre-test vs. Post-tests 1–3). Significant effects of time point show that errors were higher in Posttest 1–3 compared to the pre-test (*p*s < 0.001). In the first test of after-effects (posttest 1), there was no difference between the groups that received only physical practice of the visuomotor rotation (Obs_Post, Act+Rest, Act). However, groups that received only observational practice (Obs_Pre) or interleaved observation and physical practice (Obs_During) showed less error than the Act group (i.e., less evidence of after-effects, *p*s < 0.001).

In Posttest 2, larger after-effects were still observed for the Act group when compared to all combined practice groups (*p*s < 0.001). This pattern of results was maintained after 24 h (Post-test 3). All groups that received combined practice performed with less error than the Act group (*p*s < 0.05). The Act+Rest group did not differ from the Act group.

Effect sizes characterizing the magnitude of after-effects are presented in Table 1. All groups showed large effect sizes for all comparisons (*d*s = 1.7–3.8), with the exception of the Obs_Pre group at Post-test 1, which had only observed (*d* = 0.3). Although the size of the after-effects at Posttest 2 were generally the largest (based on effect size magnitude), sizeable after-effects persisted to Posttest 3 for all groups.

**Figure 6:**
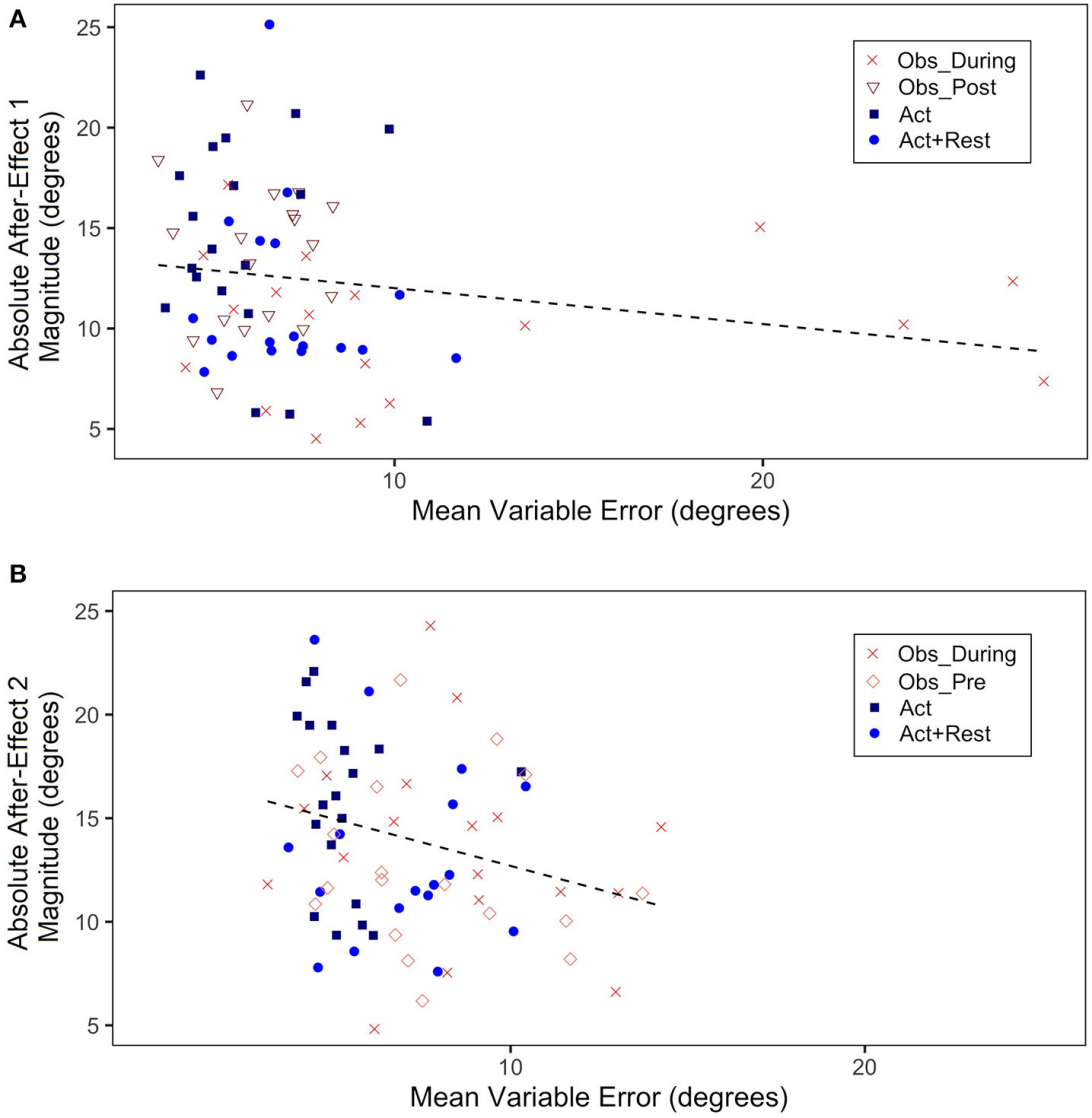
**(A)** Absolute mean after-effect magnitude (absolute value of difference of mean CE during first 5 trials of Post-test1–last 5 trials of Pre-test, in degrees) plotted as a function of mean VE during Adaptation 1 (all trials, in degrees). **(B)** Absolute mean after-effect magnitude (absolute value of difference of mean CE during first 5 trials of Post-test2–last 5 trials of Pretest, in degrees) plotted as a function of mean VE during Adaptation 2 (all trials, in degrees). Each data point represents a single participant.

We also ran correlations between VE and after-effect magnitudes (absolute values of post-test–pre-test for Adaptation 1 and 2), in view of a suspected inverse relation between between-trial variability (thought to index a sampling strategy to correctly aim to the target) and the size of after-effects, which index implicit adaptation processes. Scatterplots for Adaptation 1 (a) and 2 (b) are shown in Figure 6. What is important to note is the high variable error for the Obs_During group (the only group in Adaptation 1 to have seen demonstrations in addition to having physical practice experience). This group also showed lower magnitude of after-effects than the other groups. Although there was no significant correlation (mostly because three of the four groups had only physically practiced at this stage), there was a trend for a negative correlation, *r* (91) = −0.18, *p* = 0.12. In Adaptation 2, when two of the groups had observational practice (Obs_Pre and Obs_During), although still small, there was a significant correlation driven by the higher VEs and lower effect size magnitudes for the combined observation groups, *r*(91) = −0.25, *p* = 0.03.

### Rotation Awareness Test

On inspection of the post-experiment drawings of planned aiming trajectory to illustrate perceived magnitude of the visuomotor rotation, the Obs_During (*M* = 14.8°, *SD* = 11.1), Obs_Pre (*M* = 7.6°, *SD* = 10.6) and Obs_Post (*M* = 10.4°, *SD* = 12.5) groups, all drew aiming angles closer to the actual rotation of 30° than the two groups that only physically practiced (Act, *M* = 6.3°, *SD* = 8.9; Act+Rest, *M* = 4.3°, *SD* = 10.7).

## Discussion

We compared groups that received different types of practice, including bouts of observational practice before, after or interspersed with physical practice during acquisition of a novel visuomotor rotation task. Our aim was to evaluate if and how different schedules of observational practice influence the acquisition accuracy and implicit and explicit processes involved in adapting to new environments, in addition to the long-term retention of these acquired skills. As such, unique to our adaptation design was an investigation of the time course of these post-practice direct-effects and after-effects across practice and after a 24-h consolidation interval. Through the inclusion of physical practice and spaced-practice controls, we asked whether observational practice serves to substitute for or augment physical practice and whether the processes underpinning observational practice effects change when observation is interspersed throughout physical practice trials (potentially engaging more implicit processes associated with recalibration of sensory-motor planning processes). We showed that observational practice augmented the entire learning process, in comparison to only giving rest during observational trials, particularly when it was interspersed throughout physical practice. Observational practice served to both substitute for physical practice (with respect to adaptation time course and direct learning benefits in immediate retention tests), as well as seemingly acting as a buffer to forgetting, when comparing the magnitude of forgetting across a retention interval for the mixed observation groups in comparison to the 100% physical practice groups. We discuss these various effects and interpretations in the sections that follow.

### Direct Benefit of Observation for Adaptation and Motor Memory Consolidation

Here we showed that observational practice can substitute for physical practice to aid adaptation to novel visual feedback conditions. Participants that received an alternating schedule of 5 trials observation and 5 trials physical practice did not differ in adaptation from a group that received twice as much physical practice. They also performed with less error in adaptation practice than individuals that received the same amount of physical practice, without adjunct observational trials. There is evidence that interleaving physical practice of a new skill with short intervals of rest (some as short as 10 s) result in performance improvements over the short rest interval, termed “micro-offline gains” (Bönstrup et al., 2020, p. 1). Although our aim was not to test for potential gains from rest, here we showed that mere spacing of practice was not sufficient to aid adaptation in comparison to filling the rest intervals with observation trials. Somewhat unexpectedly, it appears that the spacing created difficulties for the rest group, causing them to show a slower rate of acquisition, more variability, and slower RTs in comparison to massed physical practice. These effects may have been related to processing demands related to memory recall and retrieval after a rest, which have been proposed as side-effects of distributed practice schedules (e.g., Küpper-Tetzel, 2014).

With respect to savings and learning, interspersing physical practice with observational practice facilitated the consolidation of memory over time. Comparisons of effect sizes showed that there was less forgetting for the Obs_During group compared to the Act group, which speaks to a more robust memory for aiming in the rotated environment as a result of the interleaved practice schedule. Indeed, when comparing across effect sizes, all combined practice groups showed less forgetting from the end of immediate Retention on day 1 to the start of Retention 2 the next day, than groups that only received physical practice. Statistical comparisons at Retention 2 showed that the interleaved combined schedule group, was still less errorful than the Act+Rest group, speaking to learning benefits associated with interspersed observational practice in comparison to interspersed rest. However, we did not see the same advantage from other combined practice schedules where observational and physical practice were given in discrete practice blocks (i.e., before or after physical practice), although neither were these groups different to the Obs_During group in Retention 2. To summarize, 100 trials of physical practice was better than only 50 trials of physical practice, but not better than 50 trials interspersed with 50 trials of observational practice. This speaks to combined practice being a suitable replacement for physical practice, at least in terms of learning accuracy.

An interleaved mixed practice schedule during practice may support better encoding of information and consolidation of motor memories than that of pure physical practice or one where physical practice and observational practice are separated. In other paradigms, similar conclusions have been made about the time sensitive nature of encoding that leads to enhanced consolidation when observation and physical practice are combined (Bove et al., 2009; Zhang et al., 2011). Both Bove and colleagues and Zhang et al. showed that duration of time between observation and execution of the same movement had a significant impact on learning. They concluded that this timing may be critical for comparisons between the sensory representations generated during observation and physical experience. In the context of an interleaved combined practice design of alternating observation and physical practice, more “switches” between each type of practice, would allow more cycles of encoding to occur [Shea et al., 2000; see also Moore et al. (2019)].

The idea that combining observation and physical practice to aid learning because of their unique benefits is not new. Others have theorized that while observational practice alone may be inferior to physical practice in terms of learning effects, the availability of physical practice attempts in a combined practice group may act to modulate a suppressed element of learning through observation (Blandin et al., 1999; Shea et al., 2000). We know that action observation plays an important role in helping to identify errors and formulate pertinent correction strategies (Lee and White, 1990; Blandin et al., 1999; Black and Wright, 2000; Hodges and Franks, 2002). In other words, the quickly acquired, though easily forgotten, explicit movement strategies, and visual representations derived from observation (Carroll and Bandura, 1990; Hodges and Franks, 2002) can be solidified (or calibrated) by the more slowly acquired implicit, motor-driven processes associated with physical practice (Gentile, 1998; Huang and Shadmehr, 2009). Although this does not mean that these processes are necessarily interactive (c.f., Mazzoni and Krakauer, 2006), there can be benefits for learning through a combined observational and physical practice approach [see also Larssen et al. (2012)].

### Observation Does Not Augment Implicit Adaptation

There was a gradual reduction in error over blocks in the after-effects trials, which has been presented by others as a signature of implicit adaptation processes (e.g., Galea et al., 2011; Kitago et al., 2013). However, the benefits associated with interleaving observation and physical practice trials did not appear to be mediated by implicit learning processes. The magnitude of after-effects, at least initially, were smaller for all combined practice groups in comparison to pure physical practice groups. In order to update/recalibrate implicit models for aiming and bring about after-effects, the dominant hypothesis is that the learner needs to be implicitly generating a feedforward prediction about the sensory consequences associated with an action (e.g., Burke et al., 2010; Wolpert et al., 2011). If there is a discrepancy between the predicted and actual sensory consequences of that movement, the resulting error will lead to recalibration of their model for aiming. It has been proposed that this sensory prediction process is only generated in the presence of a motor command and as such implicit recalibration will not occur without it (Held and Hein, 1958). In the case of observation, where no motor command is generated, such sensory error-based implicit adaptation should not occur. Indeed, the Obs_Pre group that after Adaptation 1 had only engaged in observational practice, failed to show after-effects in the first post-test [see also Ong and Hodges (2010); Ong et al. (2012); Lim et al. (2014)]. This conclusion and interpretation stands in contrast to other ideas that observational practice can lead to generation of a motor command and prediction of sensory consequences based on another's movement, leading to similar updating of internal models for aiming based on simulative mechanisms (e.g., Wolpert and Flanagan, 2001).

The result that after-effects remained small or did not increase when observation was interspersed with physical practice is in conflict with the results of Ong et al. (2012). In this previous work, direct adaptation benefits of an interleaved observation and physical practice schedule, compared to pure physical practice, were also accompanied by large after-effects. However, as detailed in the introduction, there were other group differences which may have been responsible for these effects. In addition to being prompted to engage in imagery during observation trials, the interspersed group had to both estimate hand position on their own aiming trials and that of the model on 50 trials during practice (compared to 25 trials for the other physical practice and observation-only groups). This estimation was designed to encourage prediction of sensory consequences associated with hand movements. While actual aiming accuracy improved for the interleaved group, self-estimation errors of hand trajectory remained high for both physical practice and interleaved groups. Because no feedback was provided about the accuracy of these predictions, this may have served to solidify any recalibration of the relationship between perceived position of the hand relative to the actual trajectory of the cursor, leading to large after-effects in the post-test. Due to these differences and the lower number of participants in the interleaved group in the Ong et al. (2012) study (*n* = 9 vs. *n* = 18), we are more confident in the veracity of the current data in terms of processes activated during observation trials. At least when not explicitly prompted to consider the calibration of perceived relative to actual hand position, interspersed observational practice only moderates explicitly driven processes.

The absence of evidence supporting a change in observational learning processes as a result of prior (or interspersed) physical practice experiences, is in line with a previous study (Lim et al., 2014). In this study, there were no changes in after-effects after observational practice, despite observational practice being given to individuals who had previously physically adapted, but had undergone washout trials to remove any after-effects before subsequent observation. Although there is evidence, at least at a neurophysiological level, that when we are watching others adapt we are covertly engaging processes that match those undertaken when we are actually moving (e.g., McGregor and Gribble, 2015), which is in line with the motor simulation hypothesis (Jeannerod, 2001), behaviourally at least, merely watching with the intention to learn, does not appear to be sufficient to drive the same changes which are observed through physical practice (i.e., updating of a sensory-motor map of relations between actual and perceived position of the arm).

### Competing but Complementary Processes Facilitating Visuomotor Adaptation With Combined Observational Practice

By the end of the second phase of adaptation, all groups showed evidence of after-effects, however the magnitude of these was lower for the combined practice groups compared to both physical practice groups. This moderation was not simply a result of less physical practice, since the Act+Rest group (matched for practice amount) had generally larger magnitude after-effects than these combined groups. Rather, we think these data show that the processes that support learning by observation competed with (and overrode) implicit processes driven by physical practice. While not directly tested in our study, others have investigated the competing influence of explicit and implicit processes supporting adaptation with some showing attenuation of implicit motor learning with implementation of an explicit learning strategy [see McDougle et al. (2016) for a review]. Observational learning during adaptation is thought to be supported by the formation and implementation of explicit strategies that can be later applied when the opportunity to physically perform the skill is presented (e.g., Larssen et al., 2012; Ong et al., 2012; Lim et al., 2014). In the context of the present study, these same explicit mechanisms that have been proposed to compete with implicit adaptation, could be responsible for the decreased magnitude after-effects we observed in the combined practice groups. Indeed, there are additional data in the current study to support the assumption of a more explicit-type learning engendered through combined observational and physical practice.

To further support the hypothesis that observation does not engage implicit adaptation processes, but rather works to support a more explicit method of adaptation, is provided by measures which have been considered in prior work to alert to strategy implementation, likely informed by awareness of the type of perturbation. Relatively high between trial variability in aiming has been associated with deliberate strategy implementation in response to outcome errors (Benson et al., 2011). Variable error (VE) was highest in the only observation group during Adapt 1 (Obs_During). Although VE decreased for all observation groups in the second Adaptation phase, at least until block 6, the two groups that had received observation trials had the highest mean VEs compared to physical practice only groups. Moreover, there was an inverse relation between this measure of variability and magnitude of after-effects, at least when half the participants had received observation trials in Adaptation 2.

Observation groups also drew larger rotation angles between their hand and cursor on post-experiment tests to probe awareness of the rotation, compared to physical practice groups, potentially alerting to less recalibration of hand and cursor. However, we acknowledge that being unaware of the perturbation is not a necessary condition for implicit recalibration (e.g., Modchalingam et al., 2019). Finally, reaction times (RTs) also remained high for the observation groups in both adaptation phases compared to the physical practice group without rest. Although there was no encouragement to move as fast as possible when a target appeared, RTs provide an index of planning time, which would be increased if participants had to rely on implementation of a strategy to correctly aim in contrast to adapting more implicitly. Although these measures (VE, rotation awareness, RT) only indicate that an explicit strategy was applied during the adaptation phases and do not provide direct evidence (c.f., Taylor et al., 2014; Werner et al., 2015), the wholistic picture we have based on multiple measures and assessments in our current experiment, points toward a conclusion that observation promoted adaptation via more strategic, explicit means compared to physical practice only.

## Conclusion

A combined practice schedule which comprised alternating short blocks of observation and physical practice had both short-term adaptation and longer-term consolidation benefits. These benefits were beyond what was seen for individuals who received the same amount of physical practice (without observation) and to groups that had observational practice in blocks either immediately preceding or following physical practice. Observational practice may indeed be a suitable replacement for physical practice trials, especially if provided in an interleaved as opposed to a blocked schedule. We hypothesize that this observational practice benefit is due to an enhanced awareness of the rotation through repeated observation and the development of an effective strategy to compensate for the rotation, which is facilitated when observational and physical practice trials are provided in small bouts, rather than separate blocks. Any benefits associated with combining these two types of practice did not appear to be supported by implicit adaptation mechanisms (i.e., a change to how observation trials were processed as evidenced by after-effect amplitude).

These data and in particular the acquisition benefits associated with interleaving observation with physical practice have implications for not only how we make recommendations for designing and augmenting practice, but also our understanding of processes that work to support potential benefits. In previous work, we have shown that observational practice differentially impacts on acquisition processes associated with the performance of competing skills (such as learning how to respond to clockwise and counterclockwise rotations; Larssen et al., 2012), benefiting the acquisition of both compared to just physical practice where interference between skills is shown. In the current design we expand on this conclusion, showing that observational practice also benefits the learning of a single skill, when it is provided in an alternating schedule alongside physical practice.

## Data Availability Statement

The raw data supporting the conclusions of this article will be made available by the authors, without undue reservation.

## Ethics Statement

The studies involving human participants were reviewed and approved by Behavioral Research Ethics Board, University of British Columbia, Vancouver. The patients/participants provided their written informed consent to participate in this study.

## Author Contributions

BL, DH, and NH: conceptualization of research questions, methodological design, and original draft preparation. DH and BL: data collection. SK and BL: data analyses. BL, DH, SK, and NH: writing, review, and editing. All authors contributed to the article and approved the submitted version.

## Conflict of Interest

The authors declare that the research was conducted in the absence of any commercial or financial relationships that could be construed as a potential conflict of interest.

## Footnotes

^1^Since one of the main aims of this study was to test for any moderation of after-effects associated with combined observation and physical practice, our sample size was determined based on estimated effect sizes reported in Ong et al. (2012). Based on a relatively large Group × Time interaction when comparing physical practice to an observation group and to an interleaved observation and physical practice group in tests of after-effects (Cohen's *f* = 1.5), apriori power calculations yielded a minimum sample size of 12 participants per group (power = 0.08, α = 0.05). However, due to the increase in the number of groups (5 vs. 3) and a change in design whereby after-effects were probed at 3 different time points, we were more conservative in our effect size estimate. Adjusted sample size calculations powered to detect a moderate effect (Cohen's *f* = 0.4, power = 0.08, α = 0.05) yielded a minimum sample size of *n* = 20/group.
