# Supplementary material for: Combining Observation and Physical Practice: Benefits of an Interleaved Schedule for Visuomotor Adaptation and Motor Memory Consolidation
Source: Front Hum Neurosci. 2021 Feb 4;15:614452. doi: 10.3389/fnhum.2021.614452 (PMC7890187; doi:10.3389/fnhum.2021.614452)
Supplement: Supplementary file 1 [file Table_1.docx]

**Supplementary materials**

Table S1. LME models of Mean Directional Constant Error (CE, in degrees) measured at peak movement velocity during Adaptation 1 and Adaptation 2. The effect of Group (Act, Act+Rest, Obs_During, Obs_Post or Obs_Pre), Block (2,4,6,8,10; 5 trials/block), and their interaction are reported. Act was used as the reference for Group effects, and Block 2 was used as the reference for Block effects. Estimates for fixed and random effects are reported, as well as 95% confidence intervals (CI) and p-values for fixed effects. Significant effects are reported in *italics*.

|  | **Adaptation 1**  **Mean Directional CE** | | | **Adaptation 2**  **Mean Directional CE** | | |
| --- | --- | --- | --- | --- | --- | --- |
| *Predictors* | *Estimates* | *CI* | *p* | *Estimates* | *CI* | *p* |
| (Intercept) | 15.95 | 13.24 – 18.65 | ***<0.001*** | 7.25 | 4.41 – 10.08 | ***<0.001*** |
| Group [Obs_During] | 5.47 | 1.59 – 9.36 | ***0.006*** | -0.47 | -4.54 – 3.60 | 0.822 |
| Group [Obs_Pre] | n/a | n/a | n/a | 6.65 | 2.64 – 10.67 | ***0.001*** |
| Group [Obs_Post] | -2.40 | -6.27 – 1.47 | 0.224 | n/a | n/a | *n/a* |
| Group [Act+Rest] | 5.75 | 1.84 – 9.67 | ***0.004*** | 6.03 | 1.96 – 10.10 | ***0.004*** |
| Block [4] | -4.82 | -7.55 – -2.09 | ***0.001*** | -2.71 | -5.07 – -0.34 | ***0.025*** |
| Block [6] | -9.10 | -11.83 – -6.37 | ***<0.001*** | -4.43 | -6.80 – -2.06 | ***<0.001*** |
| Block [8] | -9.34 | -12.07 – -6.62 | ***<0.001*** | -4.34 | -6.71 – -1.97 | ***<0.001*** |
| Block [10] | -10.64 | -13.37 – -7.91 | ***<0.001*** | -4.71 | -7.08 – -2.35 | ***<0.001*** |
| Group [Obs_During] * Block [4] | -2.36 | -6.28 – 1.55 | 0.237 | -0.98 | -4.38 – 2.41 | 0.570 |
| Group [Obs_Pre] * Block [4] | n/a | n/a | n/a | -1.09 | -4.44 – 2.26 | 0.524 |
| Group [Obs_Post] * Block [4] | 1.10 | -2.80 – 5.01 | 0.580 | n/a | n/a | n/a |
| Group [Act+Rest] * Block [4] | 0.38 | -3.57 – 4.34 | 0.850 | 1.17 | -2.23 – 4.57 | 0.500 |
| Group [Obs_During] * Block [6] | -2.36 | -6.29 – 1.56 | 0.237 | 1.37 | -2.03 – 4.77 | 0.431 |
| Group [Obs_Pre] * Block [6] | n/a | n/a | n/a | -2.26 | -5.62 – 1.10 | 0.187 |
| Group [Obs_Post] * Block [6] | 1.39 | -2.51 – 5.30 | 0.484 | n/a | n/a | n/a |
| Group [Act+Rest] * Block [6] | 1.55 | -2.42 – 5.51 | 0.444 | 0.25 | -3.15 – 3.66 | 0.883 |
| Group [Obs_During] * Block [8] | -6.10 | -10.04 – -2.17 | ***0.002*** | -0.20 | -3.61 – 3.21 | 0.909 |
| Group [Obs_Pre] * Block [8] | n/a | n/a | n/a | -2.75 | -6.10 – 0.61 | 0.109 |
| Group [Obs_Post] * Block [8] | 0.90 | -3.00 – 4.81 | 0.650 | n/a | n/a | n/a |
| Group [Act+Rest] * Block [8] | -0.29 | -4.24 – 3.67 | 0.887 | -1.29 | -4.69 – 2.11 | 0.458 |
| Group [Obs_During] * Block [10] | -5.59 | -9.50 – -1.67 | ***0.005*** | 1.84 | -1.56 – 5.23 | 0.289 |
| Group [Obs_Pre] * Block [10] | n/a | n/a | n/a | -3.45 | -6.80 – -0.09 | ***0.044*** |
| Group [Obs_Post] * Block [10] | 2.45 | -1.45 – 6.36 | 0.218 | n/a | n/a | n/a |
| Group [Act+Rest] * Block [10] | -0.78 | -4.74 – 3.17 | 0.698 | -1.78 | -5.18 – 1.62 | 0.306 |
| **Random Effects** | **Adaptation 1** | | | **Adaptation 2** | | |
| σ^2^ | 90.36 | | | 68.76 | | |
| τ_00_ _participant_ | 17.50 | | | 25.84 | | |
| ICC | 0.16 | | | 0.27 | | |
| N _participant_ | 73 | | | 74 | | |
| Observations | 1794 | | | 1834 | | |
| Marginal R^2^ / Conditional R^2^ | 0.196 / 0.327 | | | 0.112 / 0.355 | | |

Table S2. LME models of Mean Variable Error (VE, inter-trial standard deviation of CE in degrees) measured during Adaptation 1 and Adaptation 2. The effect of Group (Act, Act+Rest, Obs_During, Obs_Post or Obs_Pre), Block (2,4,6,8,10), and their interaction are reported. Act was used as the reference for Group effects, and Block 2 was used as the reference for Block effects. Estimates for fixed and random effects are reported, as well as 95% confidence intervals (CI) and p-values for fixed effects. Significant effects are reported in *italics*.

|  | **Adaptation 1**  **Mean VE** | | | **Adaptation 2**  **Mean VE** | | |
| --- | --- | --- | --- | --- | --- | --- |
| *Predictors* | *Estimates* | *CI* | *p* | *Estimates* | *CI* | *p* |
| (Intercept) | 7.23 | 4.85 – 9.61 | ***<0.001*** | 6.59 | 4.80 – 8.38 | ***<0.001*** |
| Group [Obs_During] | 4.32 | 0.91 – 7.73 | ***0.013*** | 3.57 | 1.00 – 6.14 | ***0.006*** |
| Group [Obs_Pre] | n/a | n/a | n/a | 4.60 | 2.07 – 7.13 | ***<0.001*** |
| Group [Obs_Post] | 1.03 | -2.39 – 4.44 | 0.555 | n/a | n/a | n/a |
| Group [Act+Rest] | -0.69 | -4.10 – 2.73 | 0.693 | 2.41 | -0.16 – 4.97 | 0.066 |
| Block [4] | -1.23 | -3.62 – 1.16 | 0.313 | -1.33 | -3.58 – 0.93 | 0.249 |
| Block [6] | -1.30 | -3.69 – 1.09 | 0.288 | -1.59 | -3.85 – 0.66 | 0.166 |
| Block [8] | -1.75 | -4.15 – 0.64 | 0.151 | -1.65 | -3.90 – 0.61 | 0.152 |
| Block [10] | -1.94 | -4.34 – 0.45 | 0.111 | -1.81 | -4.06 – 0.45 | 0.116 |
| Group [Obs_During] * Block [4] | 1.44 | -1.99 – 4.87 | 0.411 | -0.19 | -3.43 – 3.04 | 0.907 |
| Group [Obs_Pre] * Block [4] | n/a | n/a | n/a | -2.00 | -5.19 – 1.19 | 0.218 |
| Group [Obs_Post] * Block [4] | -0.17 | -3.60 – 3.26 | 0.923 | n/a | n/a | n/a |
| Group [Act+Rest] * Block [4] | 2.92 | -0.51 – 6.35 | 0.095 | -1.22 | -4.45 – 2.02 | 0.461 |
| Group [Obs_During] * Block [6] | 2.47 | -0.96 – 5.90 | 0.158 | 1.18 | -2.05 – 4.41 | 0.474 |
| Group [Obs_Pre] * Block [6] | n/a | n/a | n/a | -1.91 | -5.10 – 1.28 | 0.240 |
| Group [Obs_Post] * Block [6] | -1.10 | -4.53 – 2.33 | 0.530 | n/a | n/a | n/a |
| Group [Act+Rest] * Block [6] | 2.55 | -0.88 – 5.98 | 0.145 | -0.95 | -4.18 – 2.28 | 0.565 |
| Group [Obs_During] * Block [8] | 1.61 | -1.82 – 5.03 | 0.359 | -2.25 | -5.49 – 0.98 | 0.172 |
| Group [Obs_Pre] * Block [8] | n/a | n/a | n/a | -3.77 | -6.96 – -0.58 | ***0.021*** |
| Group [Obs_Post] * Block [8] | -0.19 | -3.62 – 3.24 | 0.914 | n/a | n/a | n/a |
| Group [Act+Rest] * Block [8] | 1.74 | -1.69 – 5.17 | 0.320 | -1.88 | -5.11 – 1.35 | 0.255 |
| Group [Obs_During] * Block [10] | 0.04 | -3.39 – 3.47 | 0.983 | -1.43 | -4.66 – 1.81 | 0.387 |
| Group [Obs_Pre] * Block [10] | n/a | n/a | n/a | -3.81 | -7.00 – -0.62 | ***0.019*** |
| Group [Obs_Post] * Block [10] | -2.03 | -5.46 – 1.40 | 0.246 | n/a | n/a | n/a |
| Group [Act+Rest] * Block [10] | 1.67 | -1.76 – 5.10 | 0.340 | -0.55 | -3.79 – 2.68 | 0.737 |
| **Random Effects** | **Adaptation 1** | | | **Adaptation 2** | | |
| σ^2^ | 14.15 | | | 12.57 | | |
| τ_00_ _participant_ | 13.88 | | | 3.30 | | |
| ICC | 0.50 | | | 0.21 | | |
| N _participant_ | 73 | | | 74 | | |
| Observations | 365 | | | 370 | | |
| Marginal R^2^ / Conditional R^2^ | 0.169 / 0.580 | | | 0.174 / 0.346 | | |

Table S3. LME models of Mean Reaction Time (RT, in ms) measured during Adaptation 1 and Adaptation 2. The effect of Group (Act, Act+Rest, Obs_During, Obs_Post or Obs_Pre), Block (2,4,6,8,10; 5 trials/block), and their interaction are reported. Act was used as the reference for Group effects, and Block 2 was used as the reference for Block effects. Estimates for fixed and random effects are reported, as well as 95% confidence intervals (CI) and p-values for fixed effects. Significant effects are reported in *italics*.

|  | **Adaptation 1**  **Mean RT** | | | **Adaptation 2**  **Mean RT** | | |
| --- | --- | --- | --- | --- | --- | --- |
| *Predictors* | *Estimates* | *CI* | *p* | *Estimates* | *CI* | *p* |
| (Intercept) | 498.66 | 289.98 – 707.35 | ***<0.001*** | 386.98 | 187.95 – 586.01 | ***<0.001*** |
| Group [Obs_During] | 205.02 | -94.38 – 504.42 | 0.180 | 432.45 | 147.01 – 717.89 | ***0.003*** |
| Group [Obs_Pre] | n/a | n/a | n/a | 253.25 | -28.37 – 534.86 | 0.078 |
| Group [Obs_Post] | 143.89 | -155.14 – 442.92 | 0.346 | n/a | n/a | n/a |
| Group [Act+Rest] | 391.77 | 91.54 – 692.01 | ***0.011*** | 322.17 | 36.73 – 607.60 | **0.027** |
| Block [4] | -48.61 | -177.40 – 80.18 | 0.459 | -4.51 | -86.54 – 77.53 | 0.914 |
| Block [6] | -98.14 | -226.93 – 30.65 | 0.135 | -33.59 | -115.85 – 48.67 | 0.423 |
| Block [8] | -98.45 | -227.24 – 30.33 | 0.134 | -16.60 | -98.84 – 65.64 | 0.692 |
| Block [10] | -127.30 | -256.08 – 1.49 | 0.053 | -35.61 | -117.64 – 46.42 | 0.395 |
| Group [Obs_During] * Block [4] | 96.04 | -88.94 – 281.02 | 0.309 | -51.09 | -168.91 – 66.72 | 0.395 |
| Group [Obs_Pre] * Block [4] | n/a | n/a | n/a | -23.88 | -140.23 – 92.47 | 0.687 |
| Group [Obs_Post] * Block [4] | -55.22 | -239.60 – 129.17 | 0.557 | n/a | n/a | n/a |
| Group [Act+Rest] * Block [4] | -44.24 | -231.07 – 142.58 | 0.643 | 23.92 | -94.04 – 141.88 | 0.691 |
| Group [Obs_During] * Block [6] | 187.43 | 2.17 – 372.69 | ***0.047*** | -39.79 | -157.76 – 78.19 | 0.509 |
| Group [Obs_Pre] * Block [6] | n/a | n/a | n/a | -42.06 | -158.72 – 74.60 | 0.480 |
| Group [Obs_Post] * Block [6] | 20.81 | -163.58 – 205.19 | 0.825 | n/a | n/a | n/a |
| Group [Act+Rest] * Block [6] | 55.29 | -132.11 – 242.70 | 0.563 | 18.11 | -100.02 – 136.24 | 0.764 |
| Group [Obs_During] * Block [8] | 232.01 | 46.25 – 417.76 | ***0.014*** | -78.88 | -197.16 – 39.41 | 0.191 |
| Group [Obs_Pre] * Block [8] | n/a | n/a | n/a | -149.90 | -266.39 – -33.40 | ***0.012*** |
| Group [Obs_Post] * Block [8] | 13.40 | -170.98 – 197.79 | 0.887 | n/a | n/a | n/a |
| Group [Act+Rest] * Block [8] | -51.78 | -238.61 – 135.04 | 0.587 | -2.99 | -120.94 – 114.95 | 0.960 |
| Group [Obs_During] * Block [10] | 220.00 | 35.02 – 404.98 | ***0.020*** | -50.43 | -168.25 – 67.38 | 0.401 |
| Group [Obs_Pre] * Block [10] | n/a | n/a | n/a | -153.09 | -269.44 – -36.74 | ***0.010*** |
| Group [Obs_Post] * Block [10] | -29.76 | -214.14 – 154.63 | 0.752 | n/a | n/a | n/a |
| Group [Act+Rest] * Block [10] | 105.70 | -81.22 – 292.63 | 0.268 | 24.70 | -93.26 – 142.66 | 0.682 |
| **Random Effects** | **Adaptation 1** | | | **Adaptation 2** | | |
| σ^2^ | 201594.94 | | | 82749.16 | | |
| τ_00_ _participant_ | 173675.05 | | | 179197.32 | | |
| ICC | 0.46 | | | 0.68 | | |
| N _participant_ | 73 | | | 74 | | |
| Observations | 1794 | | | 1834 | | |
| Marginal R^2^ / Conditional R^2^ | 0.073 / 0.502 | | | 0.086 / 0.711 | | |

Table S4. Assessment of retention savings. LME model assessment of Mean Directional Constant Error (CE, in degrees) measured at peak movement velocity comparing the effects of Group (all), Time point (3), and their interaction. The three time points included were the first block of adaptation on Day 1 (first five trials where participants physically practiced the task), Retention 1 (last 5 trials; Day 1) and Retention 2 (first 5 trials; Day 2) time points. Act was used as the reference for Group effects, and Block 1 of adaptation was used as the reference for Time point effects. Estimates for fixed and random effects are reported, as well as 95% confidence intervals (CI) and p-values for fixed effects. Significant effects are reported in *italics*.

| \|  \| **Mean Directional CE** \| \| \| \| --- \| --- \| --- \| --- \| \| *Predictors* \| *Estimates* \| *CI* \| *p* \| \| (Intercept) \| 22.89 \| 20.33 – 25.44 \| ***<0.001*** \| \| Group [Obs_During] \| -1.47 \| -5.15 – 2.21 \| 0.434 \| \| Group [Obs_Pre] \| -3.25 \| -6.88 – 0.37 \| 0.079 \| \| Group [Obs_Post] \| -2.05 \| -5.71 – 1.60 \| 0.271 \| \| Group [Act+Rest] \| -0.91 \| -4.60 – 2.79 \| 0.631 \| \| Time point [Retention 1] \| -20.66 \| -23.30 – -18.03 \| ***<0.001*** \| \| Time point [Retention 2] \| -12.74 \| -15.38 – -10.10 \| ***<0.001*** \| \| Group [Obs_During] * Time point  [Retention 1] \| 0.23 \| -3.57 – 4.04 \| 0.904 \| \| Group [Obs_Pre] * Time point  [Retention 1] \| 5.33 \| 1.59 – 9.07 \| ***0.005*** \| \| Group [Obs_Post] * Time point  [Retention 1] \| 3.12 \| -0.67 – 6.91 \| 0.107 \| \| Group [Act+Rest] * Time point [Retention 1] \| 3.37 \| -0.46 – 7.20 \| 0.085 \| \| Group [Obs_During] * Time point [Retention 2] \| -0.44 \| -4.24 – 3.37 \| 0.822 \| \| Group [Obs_Pre] * Time point [Retention 2] \| 3.59 \| -0.15 – 7.33 \| 0.060 \| \| Group [Obs_Post] * Time point [Retention 2] \| 2.31 \| -1.48 – 6.10 \| 0.232 \| \| Group [Act+Rest] * Time point [Retention 2] \| 5.14 \| 1.28 – 9.00 \| ***0.009*** \| \| **Random Effects** \| \| \| \| \| σ^2^ \| 85.65 \| \| \| \| τ_00_ _participant_ \| 14.97 \| \| \| \| ICC \| 0.15 \| \| \| \| N _participant_ \| 92 \| \| \| \| Observations \| 1359 \| \| \| \| Marginal R^2^ / Conditional R^2^ \| 0.366 / 0.460 \| \| \| |  |
| --- | --- | --- | --- | --- | --- | --- | --- | --- | --- | --- | --- | --- | --- | --- | --- | --- | --- | --- | --- | --- | --- | --- | --- | --- | --- | --- | --- | --- | --- | --- | --- | --- | --- | --- | --- | --- | --- | --- | --- | --- | --- | --- | --- | --- | --- | --- | --- | --- | --- | --- | --- | --- | --- | --- | --- | --- | --- | --- | --- | --- | --- | --- | --- | --- | --- | --- | --- | --- | --- | --- | --- | --- | --- | --- | --- | --- | --- | --- | --- | --- | --- | --- | --- | --- | --- | --- | --- | --- | --- | --- | --- | --- | --- | --- | --- | --- | --- |

Table S5. Assessment of after-effects. LME model assessment off Mean Directional Constant Error (CE, in degrees) measured at peak movement velocity, for the effect of Group (all), Time point (Pretest, Posttest 1-3), and their interaction. Act was used as the reference for Group effects, and Pretest was used as the reference for Block effects. Estimates for fixed and random effects are reported, as well as 95% confidence intervals (CI) and p-values for fixed effects. Significant effects are reported in *italics*.

|  | **Mean Directional CE** | | |
| --- | --- | --- | --- |
| *Predictors* | *Estimates* | *CI* | *p* |
| (Intercept) | -3.24 | -4.74 – -1.73 | ***<0.001*** |
| Group [Obs_During] | -0.74 | -2.90 – 1.42 | 0.500 |
| Group [Obs_Pre] | 0.49 | -1.64 – 2.62 | 0.653 |
| Group [Obs_Post] | 0.03 | -2.10 – 2.17 | 0.975 |
| Group [Act+Rest] | 0.40 | -1.74 – 2.53 | 0.716 |
| Time point [Posttest 1] | -11.21 | -12.42 – -10.00 | ***<0.001*** |
| Time point [Posttest 2] | -13.17 | -14.38 – -11.96 | ***<0.001*** |
| Time point [Posttest 3] | -13.02 | -14.23 – -11.81 | ***<0.001*** |
| Group [Obs_During] * Time point [Posttest 1] | 3.73 | 2.00 – 5.46 | ***<0.001*** |
| Group [Obs_Pre] * Time point [Posttest 1] | 10.35 | 8.64 – 12.06 | ***<0.001*** |
| Group [Obs_Post] * Time point [Posttest 1] | 0.02 | -1.72 – 1.76 | 0.982 |
| Group [Act+Rest] * Time point [Posttest 1] | 1.43 | -0.31 – 3.16 | 0.106 |
| Group [Obs_During] * Time point [Posttest 2] | 2.52 | 0.79 – 4.26 | ***0.004*** |
| Group [Obs_Pre] * Time point [Posttest 2] | 3.30 | 1.59 – 5.01 | ***<0.001*** |
| Group [Obs_Post] * Time point [Posttest 2] | 5.37 | 3.63 – 7.10 | ***<0.001*** |
| Group [Act+Rest] * Time point [Posttest 2] | 1.18 | -0.55 – 2.92 | 0.181 |
| Group [Obs_During] * Time point [Posttest 3] | 1.88 | 0.15 – 3.62 | ***0.033*** |
| Group [Pre] * Time point [Posttest 3] | 2.96 | 1.25 – 4.67 | ***0.001*** |
| Group [Post] * Time point [Posttest 3] | 1.77 | 0.01 – 3.52 | ***0.048*** |
| Group [Act+Rest] * Time point [Posttest 3] | 0.60 | -1.14 – 2.35 | 0.497 |
| **Random Effects** | | | |
| σ^2^ | 14.47 | | |
| τ_00_ _participant_ | 7.61 | | |
| ICC | 0.34 | | |
| N _participant_ | 92 | | |
| Observations | 1467 | | |
| Marginal R^2^ / Conditional R^2^ | 0.544 / 0.701 | | |
